# Iatrogenic Budd-Chiari Syndrome Secondary to Transhepatic Hemodialysis Catheterization

**DOI:** 10.7759/cureus.36433

**Published:** 2023-03-20

**Authors:** Braden G Thomas, Manuel Garza, Benjamin Warren, Matt Khosnevis, Sudha Kodali

**Affiliations:** 1 Internal Medicine, Houston Methodist Hospital, Houston, USA; 2 Gastroenterology and Hepatology, Houston Methodist Hospital, Houston, USA; 3 Pulmonary and Critical Care Medicine, Houston Methodist Hospital, Houston, USA; 4 Hepatology and Transplant Medicine, Houston Methodist Hospital, Houston, USA

**Keywords:** catheter-associated thrombosis, tunneled dialysis catheter, liver failure, hemodialysis access, hemodialysis, hepatic vein thrombosis, budd-chiari syndrome, trans hepatic venous catheterization, end stage renal disease (esrd)

## Abstract

Hepatic vein thrombosis (HVT), or Budd-Chiari syndrome, is a rare disorder resulting from the obstruction of blood flow from the liver to the inferior vena cava and eventually back to the heart. HVT can lead to extensive hepatocellular injury and portal hypertension and may require liver transplantation in patients. We present a rare cause of HVT and subsequent liver failure secondary to transhepatic venous catheterization for hemodialysis (HD) in a patient with end-stage renal disease (ESRD).

## Introduction

According to the Centers for Disease Control and Prevention data, the prevalence of end-stage renal disease (ESRD) has more than tripled from 1990 to 2018, with more than 785,000 patients in the United States alone [1]. As ESRD is a progressive disease, renal replacement must be initiated in a timely manner to avoid mortality in patients. While arteriovenous (AV) fistulas and grafts are the preferred long-term central venous access for hemodialysis (HD), other sites must be considered when these fail. In around 15% of ESRD patients with multiple access failures, tunneled dialysis catheter (TDC) placement provides alternate access sites for HD [2]. These central venous sites most commonly include the jugular, subclavian, and femoral veins [3]. However, repeat use or complications, most commonly infections, occlusions, or mechanical complications, can make even these secondary access sites less ideal [4]. In these cases, alternative and more obscure routes can be lifesaving. These routes include transhepatic venous catheterization, which can be used as a functional alternative in some ESRD patients without another accessible central venous site. However, despite being a potentially lifesaving option, transhepatic catheters are typically utilized as a last resort for vascular access due to their increased rates of catheter dysfunction, most frequently caused by thrombosis [3]. We describe the case of a patient with multiple HD access-site failures presenting with hepatic vein thrombosis (HVT), more specifically iatrogenic Budd-Chiari syndrome, and subsequent acute liver failure as a complication of transhepatic HD catheter placement.

## Case presentation

**Figure 1:**
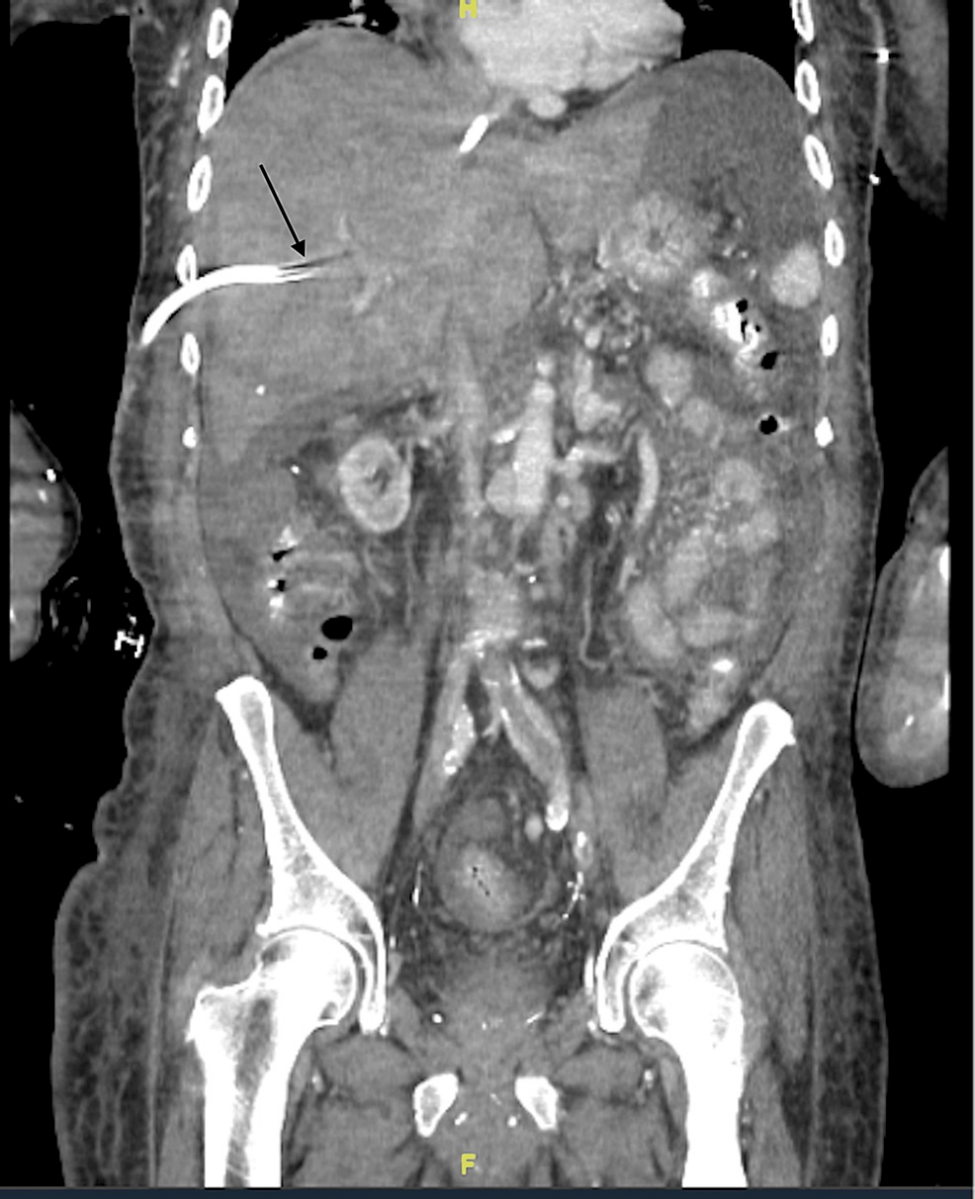
Triple-phase CT of the abdomen The image shows a diffusely enlarged and edematous liver with a lack of opacification of the hepatic veins, suggestive of hepatic vein thrombosis. Transhepatic catheter with near complete occlusion of flow, as shown by the arrow CT: computed tomography

A 68-year-old male with a past medical history of ESRD on HD, peripheral vascular disease (PVD), prior cerebrovascular accident (CVA), and deep vein thrombosis (DVT) presented from his dialysis center with a non-functioning HD catheter and hypotension. The patient notably had a transhepatic HD catheter placed one month prior to admission after the failure of HD catheters at other sites. Despite an exchange four days prior to admission, the transhepatic catheter had again ceased to function. Upon presentation, the patient had mild abdominal pain and diffuse edema. He was afebrile with a blood pressure of 97/39 mmHg, heart rate of 73 beats/minute, respiratory rate of 18 breaths/minute, and SpO_2_ of 96%. Initial labs were notable for several metabolic derangements secondary to missed HD sessions, along with a hepatocellular injury as evidenced by alanine aminotransferase (ALT) of 376 U/L and aspartate aminotransferase (AST) of 327 U/L. He underwent an uneventful transhepatic HD catheter exchange. However, the next day, the patient required ICU transfer for worsening hypotension along with increasing transaminases and a rising international normalized ratio (INR). A triple-phase CT scan of the abdomen was significant for an occluded hepatic vein (Figure 1). Doppler ultrasound demonstrated no flow through the hepatic vein. The patient was given a dose of tissue plasminogen activator (tPA) and was started on a heparin drip, which was subsequently discontinued due to hematochezia and bleeding around IV access sites. The liver function continued to worsen, with an AST of 3,946, ALT of 1,941, alkaline phosphatase (ALP) of 367, total bilirubin of 4.9, and INR of 7.1. The patient continued to need pressors and, given multiple comorbidities, was deemed a poor candidate for liver transplantation. Due to the continued worsening of his clinical status, a decision was ultimately made to pursue hospice care.

## Discussion

We presented a severe case of HVT and subsequent acute liver failure as a complication of transhepatic venous catheterization. Transhepatic venous catheters are a novel alternative in ESRD patients who do not have another central venous route accessible. It was first studied and described in 1994, in a report by Po et al., and has been further reviewed in a few additional studies since then [2,3,5]. Transhepatic catheters can be safely placed and have similar complication and infection risk profiles when compared to the more common central venous sites. However, they have been associated with a high rate of catheter thrombosis [4]. Thrombosis is a known complication of any vascular access device, but when compared to the most common venous catheter access, a transjugular catheter, the risk of thrombosis is significantly higher with a transhepatic catheter. According to one study, the overall risk of thrombosis in patients with a transhepatic catheter was 47%, as compared to 26% with jugular catheters [3,6]. While these catheters are safe to place and otherwise have minimal complications overall, the high rate of thrombosis leads to poor catheter patency, leading to missed HD sessions and further complications [4].

## Conclusions

Transhepatic catheterization, while providing a site of last resort for HD access, significantly increases the risk of venous thrombosis and subsequent complications. Hence, transhepatic catheters should be reserved for use in patients in whom all other conventional venous access sites have failed. Our patient’s vascular risk factors, including ESRD, PVD, CVA, and DVT, further increased his risk of venous thrombosis and ultimately led to acute liver failure and death. This case highlights the risk of thrombosis and compromised blood flow to the liver associated with transhepatic venous catheters, despite the procedure's utility in providing venous access for ESRD patients. Hence, it would be prudent to have a risk-benefit discussion prior to the placement of these catheters.

## References

[1] (2022). U.S. Department of Health and Human Services. Kidney disease statistics for the United States. National Institute of Diabetes and Digestive.

[2] Malviya PB, Andrews R, Ghodke A, Patel B, Reddy A (2020). Percutaneous transhepatic vein permcath: a case report. Indian J Nephrol.

[3] Şanal B, Nas ÖF, Doğan N (2016). Safety and functionality of transhepatic hemodialysis catheters in chronic hemodialysis patients. Diagn Interv Radiol.

[4] Pereira K, Osiason A, Salsamendi J (2015). Vascular access for placement of tunneled dialysis catheters for hemodialysis: A systematic approach and clinical practice algorithm. J Clin Imaging Sci.

[5] Po CL, Koolpe HA, Allen S, Alvez LD, Raja RM (1994). Transhepatic PermCath for hemodialysis. Am J Kidney Dis.

[6] Wilkin TD, Kraus MA, Lane KA, Trerotola SO (2003). Internal jugular vein thrombosis associated with hemodialysis catheters. Radiology.

